# Early development of non-hodgkin lymphoma following initiation of newer class antiretroviral therapy among HIV-infected patients - implications for immune reconstitution

**DOI:** 10.1186/1742-6405-7-44

**Published:** 2010-12-14

**Authors:** Gregory D Huhn, Sheila Badri, Sonia Vibhakar, Frank Tverdek, Christopher Crank, Ronald Lubelchek, Blake Max, David Simon, Beverly Sha, Oluwatoyin Adeyemi, Patricia Herrera, Allan Tenorio, Harold Kessler, David Barker

**Affiliations:** 1 Division of Infectious Diseases, The Ruth M. Rothstein CORE Center, 2020 W. Harrison St., Chicago, Illinois, 60612, USA; 2 Department of Medicine, Rush University Medical Center, 600 S. Paulina St., Chicago, Illinois, 60612, USA; 3 Department of Pharmacy Practice, University of Illinois at Chicago, 833 South Wood St., Illinois, 60612, USA; 4 Department of Pharmacy, The Ruth M. Rothstein CORE Center, 2020 W. Harrison St., Chicago, Illinois, 60612, USA

## Abstract

**Background:**

In the HAART era, the incidence of HIV-associated non-Hodgkin lymphoma (NHL) is decreasing. We describe cases of NHL among patients with multi-class antiretroviral resistance diagnosed rapidly after initiating newer-class antiretrovirals, and examine the immunologic and virologic factors associated with potential IRIS-mediated NHL.

**Methods:**

During December 2006 to January 2008, eligible HIV-infected patients from two affiliated clinics accessed Expanded Access Program antiretrovirals of raltegravir, etravirine, and/or maraviroc with optimized background. A NHL case was defined as a pathologically-confirmed tissue diagnosis in a patient without prior NHL developing symptoms after starting newer-class antiretrovirals. Mean change in CD4 and log_10 _VL in NHL cases compared to controls was analyzed at week 12, a time point at which values were collected among all cases.

**Results:**

Five cases occurred among 78 patients (mean incidence = 64.1/1000 patient-years). All cases received raltegravir and one received etravirine. Median symptom onset from newer-class antiretroviral initiation was 5 weeks. At baseline, the median CD4 and VL for NHL cases (n = 5) versus controls (n = 73) were 44 vs.117 cells/mm3 (p = 0.09) and 5.2 vs. 4.2 log_10 _(p = 0.06), respectively. The mean increase in CD4 at week 12 in NHL cases compared to controls was 13 (n = 5) vs. 74 (n = 50)(p = 0.284). Mean VL log_10 _reduction in NHL cases versus controls at week 12 was 2.79 (n = 5) vs. 1.94 (n = 50)(p = 0.045).

**Conclusions:**

An unexpectedly high rate of NHL was detected among treatment-experienced patients achieving a high level of virologic response with newer-class antiretrovirals. We observed trends toward lower baseline CD4 and higher baseline VL in NHL cases, with a significantly greater decline in VL among cases by 12 weeks. HIV-related NHL can occur in the setting of immune reconstitution. Potential immunologic, virologic, and newer-class antiretroviral-specific factors associated with rapid development of NHL warrants further investigation.

## Introduction

Non-Hodgkin lymphoma (NHL) is an HIV-associated malignancy that has been decreasing in incidence, ranging from approximately 1 to 3 cases per 1,000 person-years, among HIV-infected persons in the highly active antiretroviral therapy (HAART) era, representing a roughly10-fold reduction from the pre-HAART era [1-9]. The main determinants for increased risk of NHL in HIV-infected persons are prolonged immunosuppression with T-cell depletion and uncontrolled plasma HIV viremia [3,4,10-15]. Epstein-Barr virus (EBV) activation has been linked to B-cell stimulation in the most common types of HIV-related NHL, diffuse large cell non-Hodgkin and Burkitt lymphomas [10,16,17]. Upon initiation of effective HAART in patients with severe immunodeficiency, immune restoration may be adversely affected by a dysregulation of pathogen-specific immune responses, commonly referred to as immune reconstitution inflammatory syndrome (IRIS) [18,19]. B-cell NHL as a manifestation of IRIS is poorly characterized and has rarely been reported; it has mainly been recognized as NHL recurrence in patients with a previous diagnosis of NHL [20-22].

During 2006 to 2008, an unprecedented number of new antiretrovirals became available through expanded access programs (EAP) for treatment-experienced HIV-infected patients with multiple-class drug resistance. We report cases of B-cell NHL among patients without prior history of NHL diagnosed rapidly after initiating newer class antiretrovirals during the EAP study period, and examine the immunologic and virologic factors associated with potential IRIS-mediated NHL.

## Methods

Two affiliated HIV clinics in Chicago, IL participated in open-label EAP trials for etravirine (TMC125-C214), raltegravir (MK0518-023), and maraviroc (A4001050) during December 2006 to January 2008. Protocols were approved by the John H. Stroger Jr. Hospital of Cook County and Rush University Medical Center institutional review board (IRB), and all subjects provided written informed consent prior to enrollment to each EAP.

During the baseline visit, physical exam and review of systems (an inventory of systems-based symptoms) were performed and newer-class antiretrovirals were distributed with optimized background regimens. The last CD4 count and HIV RNA viral load (VL) measured in a subject before regimens including newer-class agents were initiated were considered baseline values. Subjects were evaluated for adverse events, CD4 and HIV RNA VL at weeks 4 (± 1 week) and 12 (± 2 weeks), and then every 12 weeks through 48 weeks as available.

We compared patients without NHL (controls) with those who developed NHL (cases) in a retrospective cohort study among subjects enrolled upon initiation of newer-class antiretrovirals. A NHL case was defined as a tissue diagnosis confirmed by pathologic examination and immunohistochemical staining in a patient without prior history of NHL, with symptoms recorded after starting newer-class regimens during the EAP study period. All NHL cases were reported as serious adverse events to the EAP sponsor. Differences in demographic information, including median age and sex, and baseline median values of CD4 and log_10 _VL, were calculated using a Mann Whitney test. Mean change in CD4 and log_10 _VL in NHL cases compared to controls at week 12, a time point at which values were collected among all cases, was analyzed using a 2-sided t test. The log_10 _of an undetectable HIV RNA VL (< 75 copies/ml) was set at 1.90, and at 5.70 for a VL > 500,000. Risk ratios and related p-values for NHL diagnoses associated with current antiretroviral use, including nucleos(t)ide reverse transcriptase inhibitor class, raltegravir, etravirine, maraviroc, darunavir/ritonavir, and enfuvirtide, were calculated using a Mantel-Haentzel *χ*^2 ^test, or Fisher's exact test where appropriate. The α of statistical significance was < 0.05. All analysis was performed using SPSS version 11.5 for Windows (SPSS Inc, Chicago, IL).

## Results

There were 5 new NHL cases identified among 78 total subjects enrolled during the EAP study period (mean incidence = 64.1/1000 patient-years) (Table 1). Two of these patients with NHL were screened for the raltegravir protocol MK 0518-023, though received raltegravir after the protocol closed in October 2007 upon FDA licensure. The median duration from HIV diagnosis to onset of NHL illness was 16 years. The median age of both cases and controls was 46 years (p = 1.0). Four of 5 cases were male (80%) compared to males comprising 83% (n = 60) of controls (p = 1.0). The median time from starting newer-class regimens to NHL symptom onset was 5 weeks.

**Table 1:** Clinical, immunologic, and virologic characteristics among subjects diagnosed with NHL after starting newer-class antiretroviral regimens

	Patient 1	Patient 2	Patient 3	Patient 4	Patient 5
Age (years)	46	57	47	46	43

Sex	Male	Male	Male	Male	Female

Race/Ethnicity	Black	Black	Black	Hispanic, non-Black	Black

Hepatitis B or hepatitis C coinfection	No	No	No	No	No

Year of HIV diagnosis	1996	1983	1988	1991	2002

Antiretroviral regimen before starting newer-class HAART	Tenofovir/emtricitabine, lopinavir/ritonavir	Tenofovir, lamivudine, lopinavir/ritonavir	Tenofovir/emtricitabine, zidovudine	Emtricitabine, nevirapine, saquinavir, ritonavir	Zidovudine/lamivudine, efavirenz

Newer-class HAART Regimens	Raltegravir, darunavir, ritonavir, enfurvitide	Raltegravir, tenofovir/emtricitabine, darunavir, ritonavir	Raltegravir, etravirine, tenofovir/emtricitabine, zidovudine	Raltegravir, tenofovir/emtricitabine, zidovudine, darunavir, ritonavir	Raltegravir, tenofovir/emtricitabine, darunavir, ritonavir

Date of newer- class HAART initiation	June 2007	November 2007	July 2007	October 2007	December 2007

Interval between newer- class HAART initiation and symptom onset (weeks)	3.5	3	20	6	5

Symptoms at onset of NHL	Ataxia, urinary incontinence	Left upper extremity numbness and weakness, diplopia	Left neck mass	Low back pain, weight loss	Odonophagia, fever

Baseline CD4	6	128	44	69	27

Week 4 (± 1) CD4	Not performed	Not performed	234	195	Not performed

Week 12 (± 2) CD4	15	4	250	40	33

Week 24 (± 2) CD4	Not performed	112	294	Not performed (deceased)	Not performed

Baseline HIV viral load	1,135,366	8,111	> 500,000	156,303	17,767

Week 4 (± 1) viral load	Not performed	Not performed	132	84	Not performed

Week 12 (± 2) viral load	1,356	Undetectable	Undetectable	195	Undetectable

Week 24 (± 2) viral load	Not performed	Undetectable	Undetectable	Not performed (deceased)	Not performed

Imaging findings	Brain MRI: 2.5 cm necrotic rim-enhancing lesion in the right basal ganglia with extensive vasogenic edema	Brain MRI: two enhancing cavernous lesions abutting the right and left internal carotid arteries	Neck CT scan: multiple enlarged left-sided level II to V lymph nodes with central necrosis and peripheral enhancement, lymphadenopathy in the left supraclavicular region	Chest/abdominal/pelvic CT scan: massive lymphadenopathy in the neck, left supraclavicular and paratracheal regions, bilateral hilum, retroperitoneum, paraaortic, peripancreatic, and retrograstric regions, extending into the splenic hilum and left kidney, with bilateral renal vessels displaced anteriorly. Multiple masslike bilateral pulmonary nodules	Neck CT scan: bilateral level II lymphadenopathy and a 6 mm nodule in the left apex with mild surrounding inflammation

Biopsy site and specimen	Right medial temporal brain biopsy	Left axilla lymph node excisional biopsy	Left cervical lymph node excisional biopsy	Left neck lymph node fine needle aspirate	Right lateral oropharyngeal wall biopsy via larynoscopy

Pathology	Diffuse large B-cell lymphoma	Atypical Burkitt lymphoma with translocation (8,14)	Diffuse large B-cell lymphoma	Diffuse large B-cell lymphoma	Plasmablastic large B-cell lymphoma

EBV in situ hybridization	Positive	Positive	Tissue preparation inadequate	Not performed	Positive

Chemotherapy	Yes	Yes	Yes	Yes	Yes

Outcome	Died August 2008	Survived	Survived	Died March 2008	Survived

**Figure 1:**
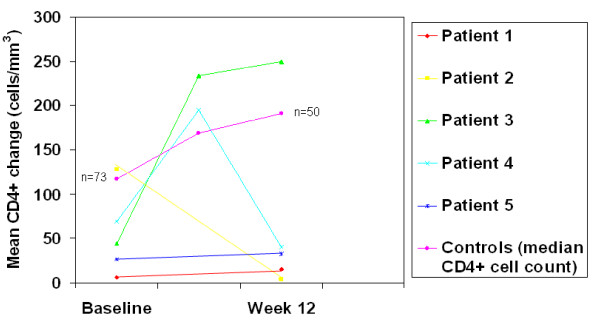
**CD4 cell count responses in NHL cases and controls after starting newer-class antiretroviral regimens**.

**Figure 2:**
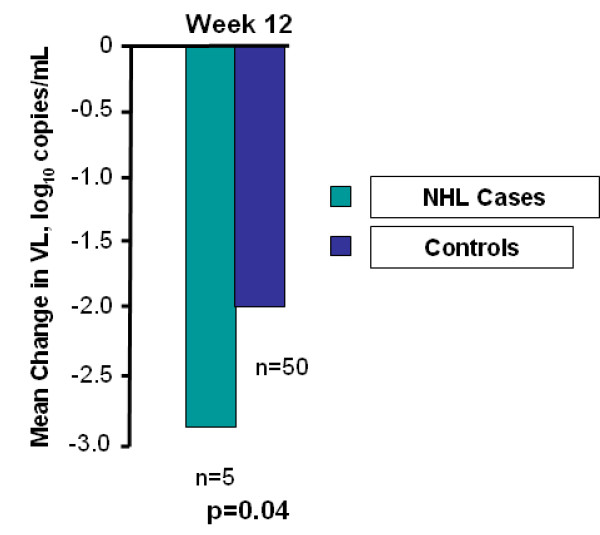
**Virologic responses in NHL cases and controls after starting newer-class antiretroviral regimens**.

At baseline, the median CD4 and VL for NHL cases (n = 5) versus controls (n = 73) were 44 vs.117 cells/mm3 (p = 0.09) and 5.2 vs. 4.2 log_10 _(p = 0.06), respectively. The mean increase in CD4 at week 12 in NHL cases compared to controls was 13 (n = 5) vs. 74 (n = 50)(p = 0.284) (figure 1). Mean VL log_10 _reduction in NHL cases versus controls at week 12 was 2.79 (n = 5) vs. 1.94 (n = 50)(p = 0.045) (figure 2). At 12 weeks, 60% (n = 3) of cases had undetectable VL compared to 64% (n = 47) of controls (p = 1.0), although the VL for patient 4 was nearly undetectable by week 12 at 84 copies/ml.

All 5 NHL cases received raltegravir. One NHL case received etravirine and no cases received maraviroc. There were no statistically significant associations between receipt of specific antiretrovirals and new NHL diagnosis (Table 2).

**Table 2:** Antiretroviral use and risk ratios for development of NHL in subjects after starting newer-class antiretroviral regimens

Antiretroviral	NHL cases (n = 5)	Controls (n = 73)	Unadjusted Odds Ratio	95% CI
Nucleos(t)ide reverse transcriptase inhibitors	100%	97%	NC	(p = 1.0)
Raltegravir	100%	86%	NC	(p = 1.0)
Etravirine	20%	53%	0.29	0.02-2.0
Maraviroc	0	11%	0	
Darunavir/ritonavir	80%	59%	2.8	0.30-26.6
Enfuvirtide	20%	4%	5.8	0.40-69.5

NC: not calculated

## Discussion

An unexpectedly high incidence of NHL, greater than 20-fold higher than currently observed in large cohort registries, was detected among treatment-experienced patients with HIV achieving high levels of virologic response with newer-class antiretrovirals. Before initiation of new HAART regimens, we observed a trend toward lower baseline CD4 cell counts and higher VLs in NHL cases, suggesting a higher degree of immunosuppression in these patients than in patients without NHL. Virologic response to newer-class HAART regimens was significantly greater at 12 weeks among all NHL cases, with a majority of these patients achieving viral suppression with undetectable VL, compared to patients without NHL. A striking characteristic in NHL cases was the rapid onset of symptomatic disease which occurred at a median of 5 weeks after starting new HAART regimens. Our analysis suggests that the development of NHL may be attributable to either severe immunosuppression in these patients or complications from IRIS.

The degree of immunosuppression in HIV infection has long been linked with risk of developing NHL [7,23,24]. In early reports assessing immunologic and virologic parameters associated with NHL, nadir CD4 count or low time-weighted mean CD4 count appeared to correlate with a high risk of NHL, which would support hypotheses that a long-term immunocompromised state may promote emergence of NHL [3,25]. Contemporary studies, however, have demonstrated that cumulative HIV viremia, particularly recent exposure to viremia, and the latest CD4 count measured before the onset of symptomatic NHL may be the most HIV-specific predictive factors for NHL oncogenesis [11,12,14,15,24,26,27]. Our analysis correlates with prior studies documenting low CD4 counts in patients with virologic failure immediately before the onset of NHL.

An important feature of our cohort was the finding of symptomatic NHL occurring in the setting of profound HIV viral load decline following initiation of HAART. This phenomenon raises the question of whether the degree and velocity of virologic response we can now achieve due to the simultaneous availability of new agents or possible unique properties of newer-class antiretrovirals may have facilitated an abnormal lymphoproliferative pathway. Clinically, in the largest cohort study of IRIS among 180 patients, the intensity of the viral load decrease within 90 days of starting HAART in antiretroviral naïve patients was the primary factor in developing IRIS. A significant association between CD4 cell count increase and IRIS was not seen until after 3 months to 9 months of HAART initiation [28]. The immunopathology of IRIS is believed to be largely mediated by a provoking antigen. In conditions in which inflammatory cell infiltration and cellular proliferation of affected tissues occur, pathogen-specific CD8 T cell responses and enhanced cytokine production appear to predominate [29-32]. In our study, EBV was identified in all NHL cases with adequate tissue for pathology examination. Control of EBV-infected B cells is achieved primarily by CD8 T cells [33,34]. Loss of EBV-specific CD8 T cell function has been associated with a decrease in total CD4 T cells in HIV-positive patients with EBV-related NHL, suggesting a lack of EBV-specific CD4 T cell help could accelerate NHL disease progression [35-41]. Loss of CD4 and CD8 memory T cells directed against latent EBV antigen (EBV nuclear antigen 1) in patients with poorly controlled HIV infection may be important components in progression to EBV-related NHL. In the Amsterdam Cohort, latent EBV-specific CD4 and CD8 T cell responses were reestablished, without changes in the EBV viral load, when effective HAART was taken longitudinally over a median of five years [42]. HIV-related NHL is characterized by high-grade tumor growth, and in recent multicenter cohort analyses incident malignancy has been assigned at ≥30 days from initiation of HAART [26]. In patients with low CD4+ counts and HIV viremia, which may promote EBV-infected B cell clonal expansion, the evolution toward EBV-related NHL may be too advanced to be modified by EBV-specific cytolytic T cell recovery upon the initiation of effective HAART. Alternatively, during an interval with rapid HIV viral decay, as can occur upon introduction of newer-class antiretroviral regimens, aberrant EBV-specific CD4, CD8, and cytokine IRIS responses may impact memory B cell stimulation, which may produce a greater risk for NHL emergence.

There were no distinct antiretrovirals that achieved statistical significance as a risk factor for developing NHL in our cohort, although the high rate of receipt of nucleos(t)ide agents and raltegravir in both groups may preclude useful comparisons. Raltegravir, however, was the only antiretroviral taken universally by all patients with NHL as part of their new HAART regimen. In the licensing trials for raltegravir in treatment-experienced patients, a disproportionately higher rate of malignancy had been reported. NHL was the most common HIV-related malignancy in these studies [43]. HIV-1 integrase inhibitors specifically inhibit the viral enzyme integrase that catalyzes strand transfer insertion of proviral DNA into the host-cell genome [44,45]. HIV-1 integrase inhibitors have a mechanism of action similar to recombination-activating genes 1 and 2 (RAG1/2), a recombinase complex fundamental to V(D)J recombination in the assemblage of human immunoglobulins, both heavy and light chains, and T cell receptors, ultimately leading to B and T cell maturation [46-50]. In vitro studies have shown HIV-1 integrase inhibitors can interfere with human DNA cleavage and disintegration activities of RAG1/2 [51]. Infrequently, RAG 1/2 has been shown to recognize and bind mistakenly to cryptic DNA sequences that are unrelated to V(D)J recombination, and these DNA elements may play a significant role in the development of lymphoid tumors [52]. RAG1/2 chromosomal transposition has also been documented in human T cells, though it appears to be an uncommon event [53]. Despite its rarity, certain oncogenic chromosomal translocations which can juxtapose immunoglobulin and T cell receptor enhancers with proto-oncogenes may be the result of RAG1/2 mediated transposition [54]. In cases suggestive of IRIS-mediated neoplasm in patients taking an integrase inhibitor as part of their HAART regimen, it is possible that any potential perturbations to the RAG1/2 system, coupled with dysfunctional T cells or enhanced cytokine responses, may be associated with the development of symptomatic malignancy.

Our study had several limitations. The analysis was prompted by reported adverse events during the EAP time period, therefore because of the nonrandomized, observational nature of the cohort, we may not have accounted for other significant risk factors for newly recognized NHL beyond HIV immunological and virologic baseline features and response rates to antiretroviral agents. NHL may have been present before patients started the newer-class HAART regimens, though physical exams and a review of systems that were obtained on all subjects upon distribution of their new antiretroviral therapy did not uncover symptomatic disease in any of the patients. At the median time of NHL symptom onset (5 weeks) after initiation of new HAART regimens, evaluable CD4 count and viral loads were either not obtained or lower due to chemotherapy for meaningful analysis in 3 of 5 NHL cases (Patients 1, 2, and 4); therefore, the implication that an IRIS possibly contributed to the early diagnosis of these malignancies is inferred primarily upon the virologic response kinetics in all cases by week 12. EBV was not identified in 2 of the 5 patients, though in both of these cases alternative pathogens were not isolated and sample limitations of the diagnostic tissue precluded evaluation for EBV markers. Lastly, the small sample size of the cohort may have led to less precision in the risk factor analysis.

In view of our results, clinicians should be vigilant in monitoring highly treatment-experienced patients for signs and symptoms of NHL as a potential IRIS complication shortly after starting potent antiretroviral therapy. Measuring elevations in circulating free immunoglobulin light chains in serum may be a sensitive marker for predicting NHL in HIV-infected patients, which may be a useful screening tool in patients at increased risk for NHL [55]. Given the similar activity of HIV integrase inhibitors to the RAG1/2 system, which is critical to B and T cell maturation, HAART regimens containing integrase inhibitors merit particular attention and further investigation as a potential novel mechanism in the development of possible IRIS-related NHL in patients with depressed immunity.

## List of Abbreviations

(NHL): non-Hodgkin lymphoma; (HAART): highly active antiretroviral therapy; (EBV): Epstein-Barr virus; (IRIS): immune reconstitution inflammatory syndrome; (EAP): expanded access programs; (IRB): institutional review board; (VL): viral load;

## Author Competing Interests

The following authors have acknowledged competing interests: GDH has served as a consultant for Gilead, MedImmune, and Genentech, received grant support from Gilead, GlaxoSmithKline, Vertex, and Merck, and received honoraria from Gilead, GlaxoSmithKline, Genentech, Merck, Sanofi Pasteur, Tibotec, Novartis, and Viiv. RH has served as a consultant and received honoraria from Gilead, and received grant support from Gilead and Tibotec. BM holds stock/stock options in GlaxoSmithKline and Pfizer, and his wife is employed by Viiv. BS has received grant support from Schering Plough and Abbott. OA has received grant support from Merck and honoraria from Abbott. AT has served as a consultant for Tibotec and received grant support from Abbott. HK has served as a consultant for Tibotec and Virco, received honoraria from Bristol-Myers Squibb, GlaxoSmithKline, and Tibotec, and holds stock in Abbott, GlaxoSmithKline, and Merck. DB has served as a consultant for Tibotec and Virco, received grant support from Merck and Pfizer, Gilead, GlaxoSmithKline, payment for development of educational presentations for Gilead. All other authors have no competing interests.

## Authors' contributions

GDH participated in the study concept and design, had full access to all of the data, carried out acquisition, analysis, and interpretation of the data, drafted the manuscript, supervised the study, and takes responsibility for the integrity of the data and the accuracy of the data analysis. SM participated in the study concept and design, carried out acquisition, analysis, and interpretation of the data, and provided administrative, technical or material support for the study. SV carried out acquisition of the data,and provided administrative, technical or material support for the study. FT carried out acquisition of the data and provided administrative, technical or material support for the study. CC carried out acquisition of the data and provided administrative, technical or material support for the study. RL carried out acquisition, analysis, and interpretation of the data, submitted critical revisions of the manuscript for important intellectual content, and provided administrative, technical or material support for the study. BM participated in the study concept and design, carried out acquisition of the data, submitted critical revisions of the manuscript for important intellectual content, and provided administrative, technical or material support for the study. DS carried out acquisition of the data and submitted critical revisions of the manuscript for important intellectual content. BS carried out acquisition of the data and submitted critical revisions of the manuscript for important intellectual content. OA carried out acquisition of the data. PH carried out acquisition of the data. AT participated in the study concept and design, carried out acquisition of the data, submitted critical revisions of the manuscript for important intellectual content, and provided administrative, technical or material support for the study. HK participated in the study concept and design, carried out analysis and interpretation of the data, submitted critical revisions of the manuscript for important intellectual content, supervised the study, and provided administrative, technical or material support for the study. DB participated in the study concept and design, carried out acquisition of the data, submitted critical revisions of the manuscript for important intellectual content, supervised the study, and provided administrative, technical or material support for the study. All authors read and approved the final manuscript.
